# Sinonasal Teratocarcinosarcoma: A Rare and Highly Aggressive Neoplasm

**DOI:** 10.7759/cureus.41396

**Published:** 2023-07-05

**Authors:** Huma Hanif, Dawood Misbah, Saad Maqsood, Sadia Iftikhar, Yasir Inam

**Affiliations:** 1 Clinical and Radiation Oncology, Shifa International Hospital Islamabad, Islamabad, PAK; 2 Clinical and Radiation Oncology, Shaukat Khanum Memorial Cancer Hospital and Research Centre, Peshawar, PAK; 3 Medical Oncology, Mater Private Hospital, Dublin, IRL; 4 Radiation Oncology, Shaukat Khanum Memorial Cancer Hospital and Research Centre, Peshawar, PAK; 5 Medicine and Surgery, Khyber Medical University, Peshawar, PAK

**Keywords:** aggressive tumor, malignant teratomas, head and neck neoplasms, radiation and clinical oncology, sinonasal teratocarcinosarcoma

## Abstract

Sinonasal teratocarcinosarcomas (SNTCSs) are malignant and highly aggressive neoplasms arising in the nasal cavity and paranasal sinuses with extension into surrounding structures and intracranial extension in few instances. They are commonly found in males (7-8 times more than in females). They pose difficulty in diagnosis due to their diverse histology. Ideal treatment modalities have not been devised yet due to the rare and highly aggressive nature of SNTCSs as shown in this case report. A 29-year-old Asian male was initially misdiagnosed on biopsy. Surgical debulking was performed initially which showed SNTCSs with radiological evidence of residual disease for which gross tumor clearance was done. He presented within a short span of a month, post re-resection with gross local recurrence. Urgent palliative radiotherapy was planned and started but unfortunately, he developed pulmonary and hepatic metastasis during radiation therapy and was commenced on palliative care only due to significant deterioration of performance status. Treatment of SNTCSs is often delayed due to their difficulty in diagnosis. Its highly aggressive nature prompts an urgent and aggressive treatment approach with adjuvant chemoradiation. Any type of adjuvant therapy is better than surgical resection only given its timely administration and close surveillance.

## Introduction

Sinonasal teratocarcinosarcomas (SNTCSs) are histologically and morphologically heterogenous malignant neoplasms. They are very rare, aggressive tumors typically arising in the nasal cavity and paranasal sinuses (ethmoid and maxillary sinus) that can also extend into the nasopharynx, anterior skull base, and rarely oral/orbital cavity [1]. In about 20% of cases, intracranial extension is also found [2]. SNTCSs comprise about 3% of all head and neck tumors and about less than 1% of all tumors [3,4]. TCS was termed “teratoid carcinosarcoma”, “teratoma”, “tetracarcinosarcoma”, or “malignant teratomas” in the past, and then in 2005, the World Health Organization approved the proper term for it as SNTCS [5]. The signs and symptoms with which SNTCSs commonly present include nasal obstruction, epistaxis, and headaches in about 75-90% of documented cases; those with intracranial obstruction might have neurological symptoms as well such as seizures, aphasia, and memory loss while other rare symptoms may include facial/ocular pain, epiphora, anosmia, odynophagia, dysphagia, expectoration of tissues, vision loss, and exophthalmos [1,6,7]. They mainly occur in the adult male population (10-82 years old have been reported with a mean age of about 50 years) with a male-to-female ratio of 7:1 - 8:1 [1,2,5,6]. SNTCSs are most often treated via surgical resection and post-operative radiotherapy but because of the rarity of these tumors and the lack of established therapies, it is challenging for the ideal treatment strategies to be identified [7]. Most often, the treatment option used is radical surgical resection followed by radiation therapy. Despite the treatment currently used, the recurrence rate is 38% and in 10.9% of cases metastasis occurs, and both recurrence and metastasis occur in 8.7% of cases [7]. SNTCSs are well known as very aggressive and rapidly progressing tumors with poor prognosis [8].

## Case presentation

**Figure 1:**
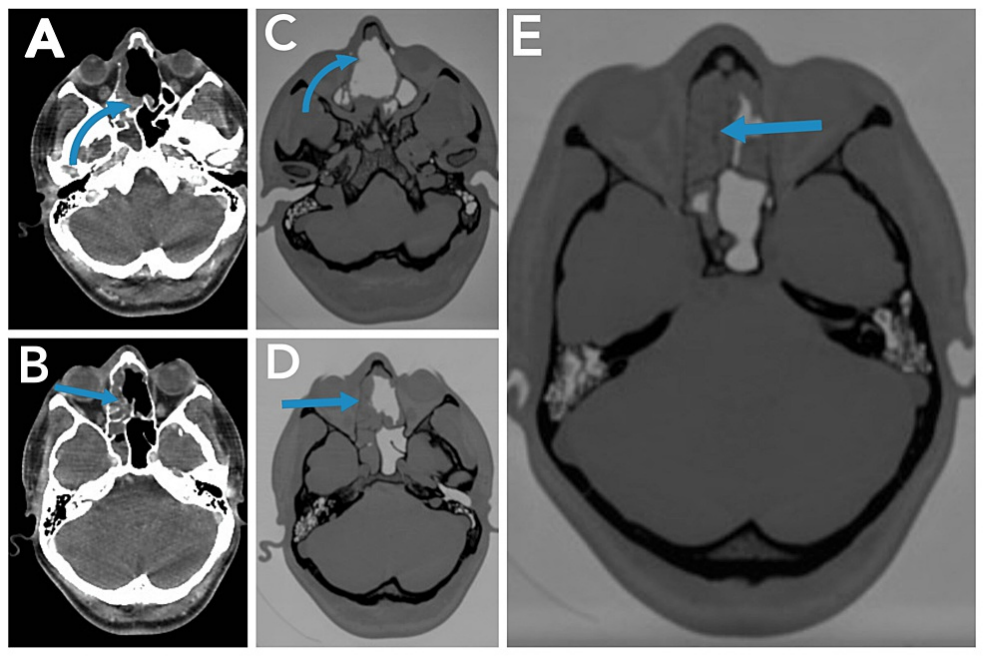
Axial sections of CT scan post-initial resection; the arrows in (A-E) show extensive post-surgical changes and/or residual disease involving the right maxillary antrum and nasal cavity

A 29-year-old male patient presented with a history of nasal obstruction and epistaxis for three months. He had no past medical history. Examination showed no remarkable findings. Initial local tissue biopsy was performed which was diagnosed as exophytic transitional cell papilloma due to inadequate samples. The patient then underwent right medial maxillectomy via the lateral rhinotomy approach, and piecemeal excision was done at the previous hospital by a head and neck surgeon and was sent for histopathology, which revealed the tumor to be teratocarsinosarcoma. So for further evaluation and treatment, he visited our hospital facility. Figure 1 shows scans taken right after the initial resection showing extensive post-surgical changes.

**Figure 2:**
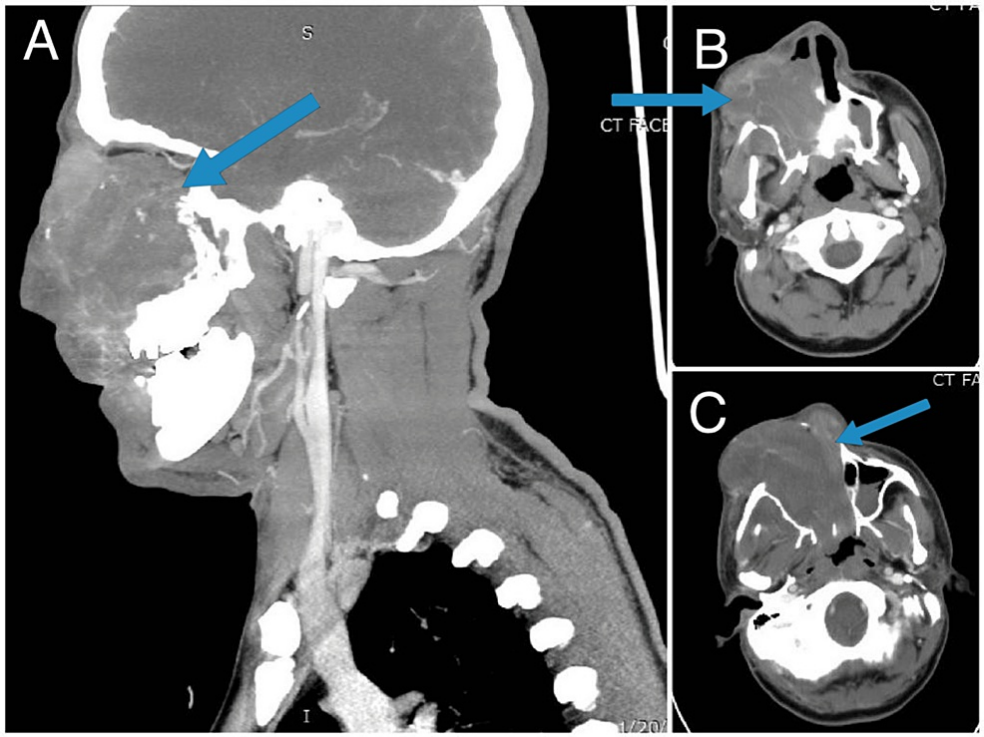
The arrows in the sagittal section in (A) and axial sections in (B) and (C) of CT scan post-re-resection show large soft tissue mass in the right maxillary sinus with pterygopalatine fossa and intraorbital extension. It also invades ethmoidal sinuses and nasal cavity with extensive right-sided neck level IIB lymphadenopathy

Post-resection staging, CT chest, abdomen, and pelvis showed no residual mass or locoregional nodal disease or distant visceral metastases. Bone scan done was also unremarkable. Clinically, the patient complained of right nasal obstruction and stuffiness while the rest of the examination findings were unremarkable. As per hospital policy, the patient discussed with the multidisciplinary tumor board who suggested to have a detailed clinical assessment and get a positron emission tomography (PET) scan for any residual disease or recurrence. The patient was physically unremarkable with good ECOG (eastern cooperative oncology group) performance status. The PET scan showed uptake in the right maxillary sinus (SUVmax 6.7) concerning residual disease, so he was referred to a head and neck surgeon and further debulking of the tumor was done via right medial maxillectomy with the Weber Ferguson approach. Operation findings showed that the tumor involved the right maxillary sinus, ethmoid sinus, and orbital periosteum along with the floor of the orbit. Gross tumor clearance was done with nasal packaging. Histopathology was consistent with teratocarsinosarcoma. One-month post-re-resection of the tumor, he developed pain and swelling of the post-operative site, right cheek, and eye and had developed new onset headaches. Upon assessment by a surgeon after a month of re-resection, early massive recurrence was detected. Urgent CT face and neck was performed which showed localized progression as shown in Figure 2.

**Figure 3:**
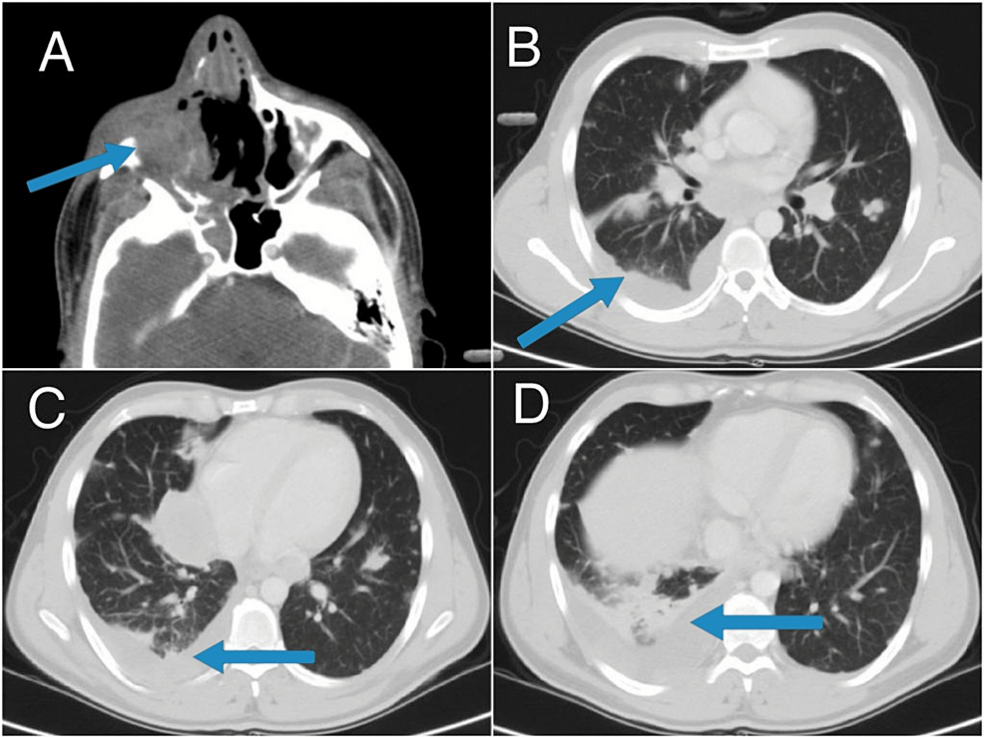
Restaging CT during radiotherapy showing via the arrow in (A) significant interval decrease in the bulky lobulated soft tissue mass with its epicenter in right maxillary sinus. The arrows in (B), (C) and (D) show progressive disease by virtue of interval development of pulmonary, pleural, and hepatic metastasis along with metastatic mediastinal lymphadenopathy

He was referred for palliative radiotherapy. Radiotherapy was initiated after assessment. High-dose palliation was planned (50 Gray doses in 25 fractions to the affected area). After three weeks (15 fractions) of radiotherapy, his re-planning CT was done which showed tumor shrinkage as shown in Figure 3. New contours were added to his planning CT. Plan was to give 50 Gray doses to the entire volume and consider a boost to primary disease if possible. Meanwhile, the patient developed dry cough and shortness of breath, so a planning CT was repeated again in the last week of radiotherapy which showed lung infiltrates and right-sided pleural effusion. Based on clinical suspicion of disease progression radiotherapy was stopped after 23 fractions and 49 Gray. Therefore, urgent restaging scans were done which revealed development of pulmonary, pleural, and hepatic metastasis along with metastatic mediastinal lymphadenopathy as well as a significant decrease of soft tissue tumor with its epicenter in the right maxillary sinus. The patient was then referred to pulmonology services for pleural effusion tapping and cytology and to the infectious disease unit for his superimposed infection, but he soon developed sepsis and remained admitted to the floor for management of sepsis; 1.4 liters of hemorrhagic pleural fluid was aspirated, and broad-spectrum antibiotics were administered. Bedside ultrasound was performed which showed collapsed consolidated lung with thick concealed multiloculated effusion. He was then referred for video-assisted thoracoscopy, but he remained unwell and on heavy oxygen support. His condition deteriorated significantly in a short span of time with a major decline in his performance status, so he was deemed unfit for chemotherapy by medical oncologists. He was referred to end-of-life hospice care only and 'Do not attempt to resuscitate' status was initiated keeping in view of his progressed unresponsive disease. He was then discharged to home on oral morphine with follow-up in a palliative pain clinic, but unfortunately, he could not make it to his follow-up appointment and died within a week.

## Discussion

**Figure 4:**
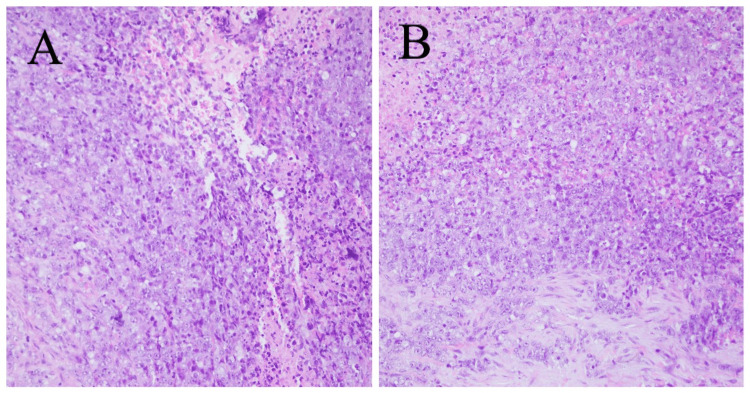
Histological pictures in (A) and (B) show sheets of large cells with predominant epithelioid morphology, large vesicular nuclei, and prominent nucleoli. A focal spindled mesenchymal component is also seen. The magnification used is 40X Sections in A and B reveal a neoplasm composed of primitive neuroepithelial elements with well-formed rosettes. Well-formed glands with atypical epithelium in myxoid stroma are seen. A piece of bone is also identified in specimens. Stains used are 1) CK: Positive in the epithelial component, 2) Synaptophysin: Focal positive in the epithelial component, 3) PLAP: Negative, 4) NSE: Focal positive is neuroepithelium, and 5) SALL4: Positive.

**Figure 5:**
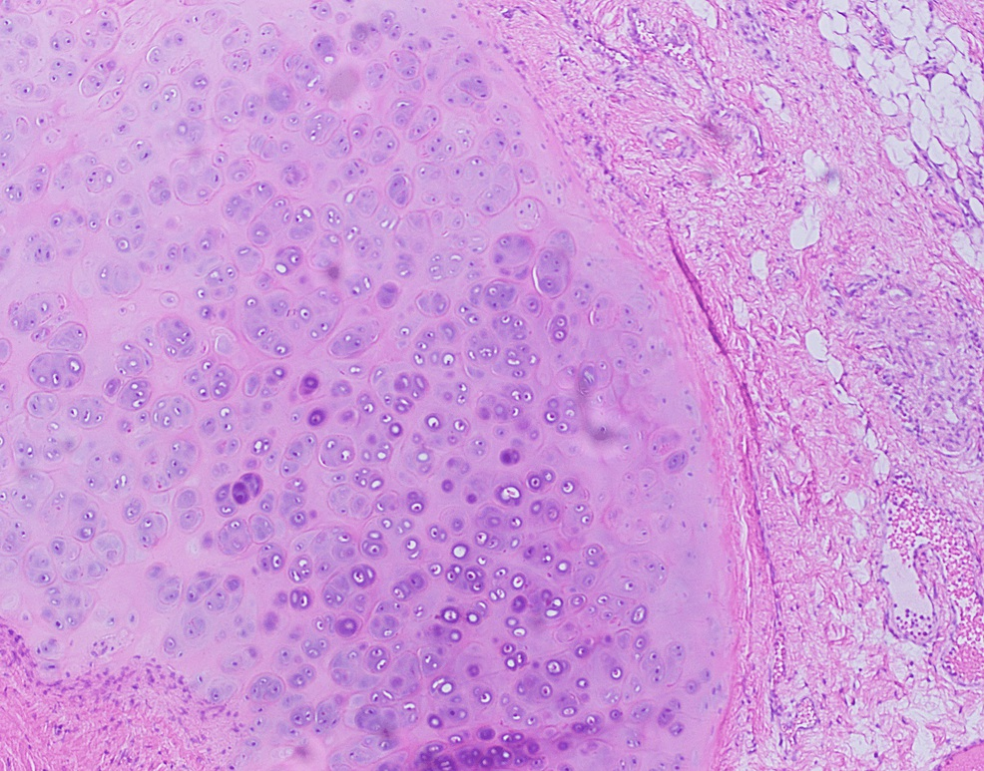
In this histological picture, cartilage can be appreciated as a part of the tumor teratomatous component. The magnification used is 40X and the stains used are CK, Synaptophysin, PLAP, NSE, and SALL4.

SNTCSs are unusual tumors, usually arising in the nasal cavity and paranasal sinuses. These tumors are mostly found by otolaryngologists during their examination of patients presenting with symptoms like epistasis, nasal congestion, and headaches [4]. They can also be confused with other tumors arising from paranasal sinuses given the histological diversity of SNTCSs so an adequate biopsy specimen is required for proper pathological examination. Simple local tissue biopsy alone is inadequate mostly. SNTCSs often metastasize to other organs in the early stage and have poor prognosis [9]. In this case, the patient was referred by an otolaryngologist after inadequate tissue sample biopsy and a wrongful initial diagnosis. They have histologic characteristics of malignant teratomas as well as carcinosarcomas with no germ cell parts and a growth pattern that includes epithelial, mesenchymal, and primitive neuroectodermal components with neural rosettes [1-3,10]. The epithelial components are highly variable that can include both benign and malignant squamous and glandular components which include columnar and cuboidal cells with or without cilia as shown in Figures 4, 5.

SNTCSs are diagnosed on the basis of malignant epithelial elements with two or more malignant mesenchymal components. These components may seem to be strange in the sinonasal tract and are more likely similar to bronchial or intestinal structures. These mesenchymal components can appear as different muscle types, cartilage, and/or bone as shown in Figure 5 [2]. “Fetal appearing” clear cell squamous epithelium, and tubular and glandular structures are also criteria for diagnosis [6]. However, this finding is not universal [7]. SNTCSs may mimic other pathologies due to the presence of multiple cell lines.

Multimodality treatment consists of surgical resection of tumor, adjuvant radiotherapy (the average dose being 54.9 Gray with range between 10-70 Gray and/or chemotherapy (most commonly cisplatin) depending on extent of disease and performance status of the patient [11]. We decided to give patient adjuvant radiotherapy (50 Gray in 25 fractions to the entire volume and treatment boost to primary disease) after re-resection of locally advanced residual disease and +/- chemotherapy.

The best five years of survival is seen by a combination of surgical intervention with radiotherapy (50%); followed by only surgery (47%). Adjuvant chemotherapy may show some survival benefits in recurrent or metastatic lesions [12]. Currently, the optimal treatment strategy of SNTCSs involves surgery along with adjuvant chemoradiotherapy. The local recurrence rate ranges from 25 to 55% with a mean of 38% [5,7], and a mean recurrence time of 21.3 months in patients followed for a mean period of 76.1 months [13]. Unfortunately, our patient's disease metastasized during adjuvant radiotherapy within a short span of 2-3 months, and due to multiple complications and a rapid decline in performance status, he was not given a trial of palliative chemotherapy. SNTCSs have a mean survival of about 1.7 years, 5-year survival of about 30-50%, and a mortality rate of 60% within three years [6,12]. Overall, the survival rate is 46% [14].

## Conclusions

SNTCSs are uncommon, highly aggressive, and generally misdiagnosed neoplasms due to their diverse histopathology. They have a high recurrence rate of 38% which prompts urgent diagnosis and aggressive treatment. Most commonly, the bimodal approach with adjuvant radiotherapy is utilized, followed by trimodality in local recurrence and distant metastasis, whereas the trimodal approach may be more effective than the bimodal approach in controlling local recurrence and metastasis, and for managing SNTCSs, the surgery alone is not as effective as any form of adjuvant therapy given its initiation on time and timely diagnosis.
